# Preoperative detection of KRAS mutated circulating tumor DNA is an independent risk factor for recurrence in colorectal cancer

**DOI:** 10.1038/s41598-020-79909-4

**Published:** 2021-01-11

**Authors:** Yuki Nakamura, Shozo Yokoyama, Kenji Matsuda, Koichi Tamura, Yasuyuki Mitani, Hiromitsu Iwamoto, Yuki Mizumoto, Daisuke Murakami, Yuji Kitahata, Hiroki Yamaue

**Affiliations:** Second Department of Surgery, School of Medicine, Wakayama Medical University, 811-1 Kimiidera, Wakayama, 641-8510 Japan

**Keywords:** Cancer, Tumour biomarkers, Predictive markers

## Abstract

Preoperative ctDNA status in relation to recurrence in cases of CRC remains unclear. We examined preoperative ctDNA detection by targeting KRAS gene mutations as a predictive marker for recurrence after CRC surgery. We measured the preoperative KRAS mutated ctDNA status and analyzed the correlation with clinicopathologic features of 180 patients that underwent surgery for CRC. We studied the association between preoperative KRAS mutated ctDNA and postoperative recurrence in patients (n = 150) that underwent radical surgery. KRAS mutated ctDNA was detected in 59 patients (32.8%). Median mutant allele frequency of KRAS in ctDNA was 0.20%. KRAS status in ctDNA and lymph node metastasis and distant metastasis were not significantly different. Among patients that underwent radical resection, recurrence occurred in 21 (14.0%, median follow-up 24 months). In Kaplan–Meier analysis, preoperative detection of KRAS mutated ctDNA was associated with inferior recurrence-free interval (RFI) (*p* = 0.002) and recurrence-free survival (RFS) (*p* = 0.025). In a multivariate Cox proportional hazards model, preoperative detection of KRAS mutated ctDNA was an independent factor related to both RFI (HR = 3.08; *p* = 0.012) and RFS (HR = 2.18; *p* = 0.044). Preoperative measurement of KRAS mutated ctDNA could be useful to decide postoperative treatment.

## Introduction

Colorectal cancer (CRC) is one of the most common types of cancer. It is important to identify predictive factors for recurrence to manage CRC patients. Adjuvant chemotherapies have been used in stage III CRC and stage II cases judged to have a high risk of recurrence based on clinicopathologic features, such as poorly differentiated tumor, vascular, lymphatic or perineural invasion, tumor depth of T4, lymph nodes sampling < 12, or clinical presentation with intestinal occlusion or perforation^1–6^. CRC treatment outcomes have improved due to recent advances in medical technology, but determining treatment strategies for postoperative recurrence is often difficult.

In recent years, circulating tumor DNA (ctDNA) has attracted attention as a predictor of postoperative recurrence. It is a fraction of cell-free DNA (cfDNA), which is derived from cancer cells and contains tumor specific DNA mutations^7–9^. ctDNA levels in plasma are typically low, but recent technological advancements, such as digital droplet PCR (ddPCR) and next generation sequencing (NGS) platforms, have enabled detection of ctDNA in frequencies as low as 0.01%^10^. The presence of postoperative circulating tumor DNA (ctDNA) has been demonstrated to be associated with recurrence after CRC surgery^11–13^. However, the relationship in CRC between preoperative ctDNA status and recurrence is unclear. Meanwhile, the presence of preoperative ctDNA has been reported to be associated with recurrence in other cancers, such as pancreatic cancer and breast cancer^14–16^. The detection rate of ctDNA has been shown to decrease after surgery^13^, so examining preoperative ctDNA status may help to distinguish more cases considered to be at high risk of recurrence.

In CRC, KRAS gene is mutated in about 40% of cases, mostly appearing in segment in exon 2 (codon 12 and 13). Point mutations in the KRAS gene have been reported to occur early in the carcinogenic process and are detected at the same frequency in tissue biopsy, regardless of the cancer stage^17,18^. KRAS exon 2 mutations have been reported to be associated with recurrence and poor prognosis after CRC surgery^18–20^. Therefore, we focused on KRAS mutations in ctDNA, but not ctDNA with other mutations. ctDNAs have various mutations including KRAS, BRAF, NRAS, PIK3CA and so on. It has been shown that KRAS and BRAF mutations are mutually exclusive^18,21^. ctDNA without KRAS mutation may contain BRAF mutation such as V600E.

In this study, we measured the preoperative KRAS mutated ctDNA and evaluated any association with clinicopathologic factors. We also evaluated the usefulness of preoperative KRAS mutated ctDNA detection as a predictive marker of recurrence after CRC surgery.

## Results

### Mutant and wild-type KRAS in cfDNA detected by ddPCR

**Figure 1:**
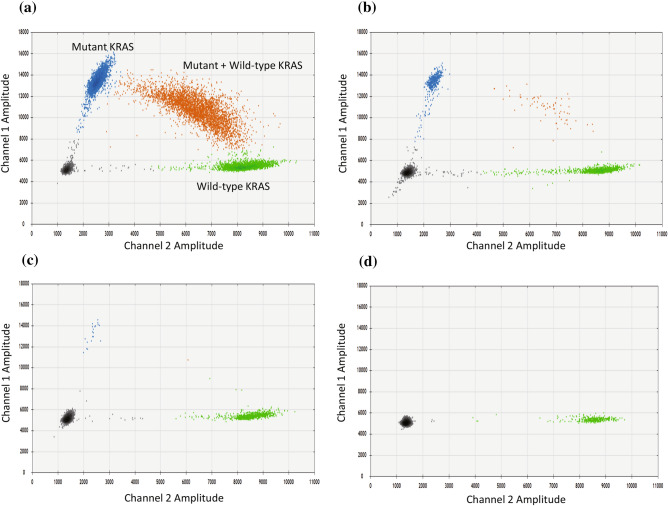
Detection of KRAS mutated ctDNA by ddPCR. (**a**–**c**) KRAS mutated ctDNA positive, (**d**) KRAS mutated ctDNA negative. Each cluster on the plot represents as follows: black cluster, negative droplets; green cluster, wild-type KRAS positive droplets; blue cluster, mutant KRAS positive droplets; orange cluster, both wild-type and mutant KRAS positive droplets.

Mutant and wild-type KRAS ctDNA were shown separately by the Quantasoft software Ver1.7.4 (Bio-Rad), such as shown in Fig. 1. The ratio of positive drops for the mutant and/or the wild-type allele was calculated as mutant allele frequency (MAF), and 0.02% was set as the lower limit of ctDNA detection, as previously reported^22^.

### KRAS mutation in ctDNA and lymph node and distant metastasis

Among 180 patients, 59 were positive for KRAS mutation in ctDNA (32.8%). The median MAF of KRAS in ctDNA was 0.20% (range 0.04–68.99%). Patient demographic data and KRAS status in ctDNA by clinicopathologic factors are shown in Table 1. The frequency of KRAS mutation positive in ctDNA was higher in cases with histological types other than well-differentiated, in cases with invasion depth of T3 or T4, cases with lymphatic invasion, cases with venous invasion, and stage IV cases. However, multivariate analysis did not reveal significant difference in these factors. Subsequently, we investigated the relationship between KRAS status in ctDNA and lymph node metastasis and with distant metastasis (Table 2). Factors such as invasion depth of T3 or T4, lymphatic invasion, venous invasion and positivity for preoperative serum CEA had correlation to lymph node and distant metastasis. Regarding KRAS status in ctDNA, cases with preoperative KRAS mutation positive for ctDNA tended to have lymph node metastasis and distant metastasis. Multivariate analysis showed venous invasion was significantly related to lymph node metastasis (*p* = 0.011), but there were no significant differences between KRAS status in ctDNA and lymph node metastasis or distant metastasis (Table 3).

**Table 1:** Clinicopathologic variables for KRAS status in ctDNA.

	Total (N = 180)	Positive (N = 59)	Negative (N = 121)	*p* value
**Age**				
≥ 66	133	45	88	0.611
< 66	47	14	33	
**Gender**				
Male	100	33	67	0.943
Female	80	26	54	
**Tumor site**				
Colon	140	45	95	0.734
Rectum	40	14	26	
**Differentiation**				
Well	57	12	45	0.027
Others	122	46	76	
**Depth**				
T3, 4	125	49	76	0.006
T1, 2	55	10	45	
**Lymphatic invasion**				
Present	58	25	33	0.036
Absent	117	32	85	
**Venous invasion**				
Present	87	37	50	0.005
Absent	88	20	68	
**TNM classification**				
Stage ≤ III	154	46	108	0.043
Stage IV	26	13	13	
**Preoperative serum CEA**				
≥ 5.0	71	29	42	0.063
< 5.0	109	30	79	

*Well* well differentiated adenocarcinoma; *others* moderately differentiated, poorly differentiated, mucinous and papillary adenocarcinoma.

**Table 2:** Univariate analysis of lymph node metastasis and distant metastasis.

Variable	Lymph node metastasis			Distant metastasis		
	Present	Absent	*p* value	Present	Absent	*p* value
**Age**						
≥ 66	60	73	0.340	21	112	0.388
< 66	25	22		5	42	
**Gender**						
Male	43	57	0.205	11	89	0.142
Female	42	38		15	65	
**Tumor site**						
Colon	63	77	0.264	21	119	0.692
Rectum	22	18		5	35	
**Differentiation**						
Well	19	38	0.013	7	50	0.657
Others	65	57		18	104	
**Depth**						
T3, 4	73	52	< 0.001	25	100	0.001
T1, 2	12	43		1	54	
**Lymphatic invasion**						
Present	38	20	< 0.001	12	46	0.013
Absent	42	75		9	108	
**Venous invasion**						
Present	56	31	< 0.001	17	70	0.002
Absent	24	64		4	84	
**Serum CEA**						
≥ 5.0	45	26	< 0.001	19	52	< 0.001
< 5.0	40	69		7	102	
**KRAS mutated ctDNA**						
Positive	34	25	0.051	13	46	0.043
Negative	51	70		13	108	

*Well* well differentiated adenocarcinoma; *others* moderately differentiated, poorly differentiated, mucinous and papillary adenocarcinoma.

**Table 3:** Multivariate analysis of lymph node metastasis and distant metastasis.

Variable	Lymph node metastasis			Distant metastasis		
	HR	95% CI	*p* value	HR	95% CI	*p* value
Differentiation (others)	1.55	0.71–3.39	0.271	–	–	–
Depth (T3, 4)	2.16	0.93–5.01	0.074	4.09	0.47–35.90	0.204
Lymphatic invasion	2.05	0.98–4.26	0.055	1.81	0.67–4.91	0.244
Venous invasion	2.59	1.25–5.37	0.011	2.33	0.67–8.06	0.182
Serum CEA (≥ 5.0)	1.63	0.80–3.34	0.180	2.29	0.82–6.40	0.114
KRAS mutated ctDNA	1.11	0.54–2.30	0.772	1.68	0.63–4.47	0.301

*HR* hazard ratio; *CI* confidence interval; others moderately differentiated, poorly differentiated, mucinous and papillary adenocarcinoma; *CEA* carcinoembryonic antigen.

### Preoperative KRAS mutation in ctDNA and recurrence

We prospectively examined the association between preoperative KRAS status in ctDNA and recurrence in 150 cases, excluding 26 cases with synchronous distant metastasis, three cases without distant metastasis who could not undergo radical resection, and one case of hospital death due to pneumonia and renal failure a month after surgery. Recurrence was diagnosed by imaging examinations. Regarding adjuvant chemotherapy, capecitabine single agent or capecitabine plus oxaliplatin was generally given to stage III and high risk stage II CRC patients in our department. In this study, the use of adjuvant chemotherapy was decided by the treating oncologist who was blinded to the ctDNA status. Capecitabine single agent was given to 32 patients and capecitabine plus oxaliplatin was given to 19 patients. Fifteen of 38 ctDNA positive patients and 36 of the 73 ctDNA negative patients received adjuvant chemotherapy in stage II and III CRC patients.

**Figure 2:**
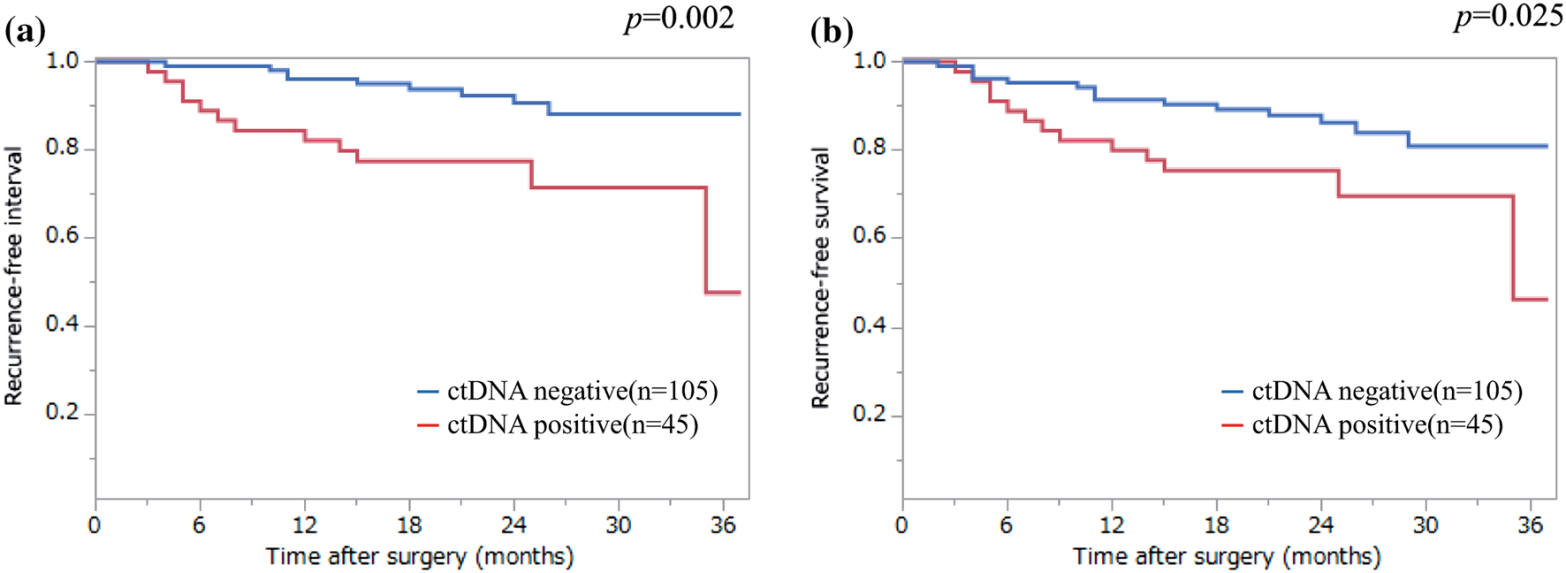
Kaplan–Meier analysis of recurrence-free interval (**a**), and recurrence-free survival (**b**), according to preoperative KRAS status in ctDNA.

Among the 150 patients with stage III or lower stage CRC that underwent radical resection, recurrence occurred in 21 patients (14.0%), including 12 of the 45 patients with detectable KRAS mutations in ctDNA (26.7%) and 9 of the 105 patients without such mutations (8.6%). Fifteen of the 150 patients (10.0%) died before median follow-up of 24 months (range 12–37 months). Patients with preoperative detectable KRAS mutations in ctDNA had an increased risk of recurrence relative to those without them (*p* = 0.003). Kaplan–Meier analysis showed that preoperative detection of KRAS mutant ctDNA was associated with inferior recurrence-free interval (RFI) (*p* = 0.002) (Fig. 2a) and recurrence-free survival (RFS) (*p* = 0.025) (Fig. 2b). In a multivariate Cox proportional hazards model, preoperative detection of KRAS mutated ctDNA was the significant factor correlated to both RFI (HR = 3.08; p = 0.012) and RFS (HR = 2.18; *p* = 0.044) (Tables 4 and 5).

**Table 4:** Univariate and multivariate analysis of recurrence-free interval.

Variable	Univariate analysis			Multivariate analysis		
	HR	95% CI	*p* value	HR	95% CI	*p* value
Age (≥ 66)	1.32	0.52–4.06	0.576			
Gender (female)	1.57	0.66–3.77	0.304			
Tumor site (colon)	1.77	0.60–7.59	0.329			
Differentiation (well)	1.07	0.41–2.58	0.878			
Depth (T3, 4)	2.33	0.86–1.16	0.101			
Lymphatic invasion	1.70	0.69–4.03	0.237			
Venous invasion	2.83	1.17–7.52	0.021	1.50	0.59–4.19	0.400
Lymph node metastasis	5.20	2.03–15.91	< 0.001	4.06	1.53–12.76	0.004
Serum CEA (≥ 5.0)	2.44	1.03–5.99	0.044	1.68	0.70–4.21	0.245
CCI (≤ 1)	1.46	0.42–9.18	0.591			
Adjuvant chemotherapy	1.40	0.57–3.33	0.447			
KRAS mutated ctDNA	3.58	1.51–8.79	0.004	3.08	1.29–7.63	0.012

*HR* hazard ratio; *CI* confidence interval; *Well* well differentiated adenocarcinoma; *CEA* carcinoembryonic antigen; *CCI* Charlson comorbidity index.

**Table 5:** Univariate and multivariate analysis of recurrence-free survival.

Variable	Univariate analysis			Multivariate analysis		
	HR	95% CI	*p* value	HR	95% CI	*p* value
Age (≥ 66)	1.90	0.78–5.65	0.168			
Gender (female)	1.63	0.78–3.49	0.195			
Tumor site (colon)	1.76	0.68–5.99	0.267			
Differentiation (well)	1.19	0.53–2.52	0.668			
Depth (T3, 4)	2.01	0.86–5.45	0.109			
Lymphatic invasion	1.08	0.46–2.32	0.856			
Venous invasion	1.83	0.87–3.97	0.114			
Lymph node metastasis	2.47	1.17–5.44	0.018	2.38	1.13–5.25	0.023
Serum CEA (≥ 5.0)	1.59	0.74–3.37	0.226			
CCI (≤ 1)	0.92	0.36–3.14	0.884			
Adjuvant chemotherapy	1.04	0.46–2.20	0.929			
KRAS mutated ctDNA	2.28	1.07–4.80	0.034	2.18	1.02–4.61	0.044

*HR* hazard ratio; *CI* confidence interval; *Well* well differentiated adenocarcinoma; *CEA* carcinoembryonic antigen; *CCI* Charlson comorbidity index.

## Discussion

KRAS mutation in CRC primary lesions were reported to be associated with a high risk of postoperative recurrence^18–20^. We demonstrated that preoperative KRAS mutation in ctDNA is an independent risk factor for recurrence in patients with CRC. Although KRAS mutation in ctDNA may be detected in plasma without malignancy, it is extremely rare. Hence, preoperative measurement of KRAS mutation in ctDNA could reflect the primary lesion^23,24^. The concordance rate of KRAS status between cancer tissue and ctDNA has been reported as 75–96%^25–27^, because the measurement of gene mutations in tissue has a problem of heterogeneity. Meanwhile a previous study of pancreatic cancer showed that mutant KRAS in plasma was significantly associated with recurrence and prognosis, but not in tumor tissue samples^27^. The measurement of KRAS mutation in ctDNA or in primary tumors may reflect different aspects of CRC.

In recent years, several reports have demonstrated “postoperative” detectable ctDNA as a predictive factor for recurrence^11–13^. However, the detection rate of ctDNA has been reported to decrease after surgery. Reinert et al. reported that in stage I-III CRC, the detection rate of ctDNA decreased from 88.5% preoperatively to 10.6% postoperatively^13^. If ctDNA mutations in “postoperative” plasma samples after radical resection can be detected, it may indicate remnant cancer cells resulting in recurrence and poor prognosis. However, if patient has a small amount of cancer cells after radical surgery, ctDNA mutations could not be detected. Preoperative KRAS mutations in ctDNA reflecting mutations of primary lesion may present malignant phenotype of primary tumor. Therefore, we analyzed “preoperative” ctDNA mutations, but not postoperative mutations in the current study. A comparison between preoperative and postoperative ctDNA may provide more information regarding recurrence and prognosis. Further investigation is needed to address this issue. In addition, preoperative ctDNA positive cases were reported to have a high risk of recurrence in localized pancreatic cancer, even if postoperative ctDNA becomes negative^15^. We showed that preoperative KRAS mutation in ctDNA is associated with recurrence of CRC. Preoperative detection of KRAS mutated ctDNA may provide adequate postoperative screening and appropriate postoperative adjuvant chemotherapy. In the current study, the significance of preoperative ctDNA measurement to determine the indication for adjuvant chemotherapy was not clarified due to a small number of patients. Clinical trial is needed to address this issue.

Circulating tumor DNA is a novel means of detecting early phase CRC recurrence. Postoperative detection of ctDNA in stage II or III CRC reflected minimal residual disease and predicted recurrence^11,12^. It was measured in these reports by NGS, but clinical application was very costly. ddPCR has been reported to measurable at a lower cost and in a shorter time, yet with higher sensitivity than NGS^26^. In the current study, KRAS mutated ctDNA measured by ddPCR was significantly correlated with recurrence of CRC and was an independent risk factor for recurrence of CRC.

Our study has several important limitations; only a comparatively small number of patients that had recurrent CRC were included, and the study was of explorative design and there was no validation cohort. Further investigations are required to address these issues.

In conclusion, the presence of KRAS mutated ctDNA before surgery was significantly associated with recurrence after radical resection in cases of CRC. Preoperative KRAS mutated ctDNA measurement was suggested to be a potentially useful biomarker to predict postoperative recurrence. Recurrence may be reduced by administering adjuvant chemotherapy to ctDNA positive patients.

## Methods

### Patients

In this study, we investigated the relationship between KRAS status in ctDNA, lymph node metastasis, distant metastasis and clinicopathologic factors including age, gender, tumor site, differentiation, tumor depth, lymphatic invasion, venous invasion, preoperative serum CEA value, co-morbidity and adjuvant chemotherapy. In order to consider the effect of co-morbidity on recurrence and prognosis, we evaluated by using the Charlson Comorbidity Index (CCI)^28,29^.

Enrolled in this study were 183 patients with CRC that underwent surgery at the Second Department of Surgery, Wakayama Medical University, between April 2017 and December 2018. We excluded patients that received preoperative treatment, such as chemotherapy, radiotherapy or endoscopic resection. We also excluded cases diagnosed with other primary cancers and cases with other tumors found by preoperative imaging examinations. In addition, in three cases, surgery was performed for preoperative clinical diagnosis of CRC, and the patients were not diagnosed with adenocarcinoma by postoperative pathological diagnosis, and these were also excluded from statistical analyses. All research was performed in accordance with relevant guidelines/regulations. This study was approved by the Wakayama Medical University Human Ethics Review Committee (Approval Number 1949) and informed consent was obtained from all included patients.

### Blood sample collection and extraction of cfDNA

Just before the start of surgery, 5 mL blood samples were obtained in EDTA tubes from each patient and centrifuged at 1900*g* for 10 min within 2 h after collection. Plasma was collected and stored at − 80 °C until use. After thawing plasma samples, they were centrifuged at 16,000*g* for 10 min. cfDNA was extracted from 2 mL of plasma using the QIAamp Circulating Nucleic Acid Kit (Qiagen) according to the manufacturer’s instructions. Samples were eluted in 75 μL elution buffer and cfDNA was frozen at − 80 °C until analysis. Blood for CEA was collected at the first visit and measured within 2 h.

### Detection of KRAS mutated ctDNA

KRAS mutations in ctDNA was analyzed by ddPCR. QX200 Droplet Digital PCR system (Bio-Rad) and ddPCR KRAS multiplex assays including G12A, G12C, G12D, G12R, G12S, G12V, G13D mutations (Bio-Rad) were used according to the manufacturer’s protocols. A reaction volume of 20 µL including 8 µL of cfDNA was used as a template for each PCR. Droplets were generated using the QX200 droplet generator (Bio-Rad) and PCR reaction was performed in a C1000 Touch Thermo Cycler (Bio-Rad) under the following conditions: 95 °C for 10 min, 40 cycles of 94 °C for 30 s and 55 °C for 1 min, and 98 °C for 10 min. Data analysis were performed using the Quantasoft software Ver1.7.4 (Bio-Rad).

### Statistics

Statistical analysis was performed using JMP ver. 14.1.0 (SAS Institute). Differences between groups were determined using Pearson’s chi-squared test to compare categorical variables as appropriate. Factors with *p* < 0.10 on univariate analysis were analyzed by multivariate logistic regression, and an odds ratio with a 95% confidence interval was calculated for each factor. The Kaplan–Meier method was used to estimate recurrence-free interval (RFI) and recurrence-free survival (RFS), and the log-rank test was used to determine the statistical significance. Cox proportional hazards model was used to assess the risk ratio under simultaneous contributions from several covariates. Final statistical results were considered significant at *p* < 0.05.

## Data Availability

The datasets generated and analyzed during the current study are available from the corresponding author on reasonable request.

## References

[1] Andre T (2004). Oxaliplatin, fluorouracil, and leucovorin as adjuvant treatment for colon cancer. N. Engl. J. Med..

[2] Haller DG (2011). Capecitabine plus oxaliplatin compared with fluorouracil and folinic acid as adjuvant therapy for stage III colon cancer. J. Clin. Oncol..

[3] Twelves C (2005). Capecitabine as adjuvant treatment for stage III colon cancer. N. Engl. J. Med..

[4] Grothey A (2018). Duration of adjuvant chemotherapy for stage III colon cancer. N. Engl. J. Med..

[5] Benson AB (2004). American Society of Clinical Oncology recommendations on adjuvant chemotherapy for stage II colon cancer. J. Clin. Oncol..

[6] Schmoll HJ (2012). ESMO Consensus Guidelines for management of patients with colon and rectal cancer. A personalized approach to clinical decision making. Ann. Oncol..

[7] Jahr S (2001). DNA fragments in the blood plasma of cancer patients: Quantitations and evidence for their origin from apoptotic and necrotic cells. Cancer Res..

[8] Diaz LA, Bardelli A (2014). Liquid biopsies: Genotyping circulating tumor DNA. J. Clin. Oncol..

[9] Bettegowda C (2014). Detection of circulating tumor DNA in early- and late-stage human malignancies. Sci. Transl. Med..

[10] Beije N (2016). Somatic mutation detection using various targeted detection assays in paired samples of circulating tumor DNA, primary tumor and metastases from patients undergoing resection of colorectal liver metastases. Mol. Oncol..

[11] Tie J (2016). Circulating tumor DNA analysis detects minimal residual disease and predicts recurrence in patients with stage II colon cancer. Sci. Transl. Med..

[12] Tie J (2019). Circulating tumor DNA analyses as markers of recurrence risk and benefit of adjuvant therapy for stage III colon cancer. JAMA Oncol..

[13] Reinert T (2019). Analysis of plasma cell-free DNA by ultradeep sequencing in patients with stages I to III colorectal cancer. JAMA Oncol..

[14] Groot VP (2019). Circulating tumor DNA as a clinical test in resected pancreatic cancer. Clin. Cancer Res..

[15] Lee B (2019). Circulating tumor DNA as a potential marker of adjuvant chemotherapy benefit following surgery for localized pancreatic cancer. Ann. Oncol..

[16] Garcia-Murillas I (2019). Assessment of molecular relapse detection in early-stage breast cancer. JAMA Oncol..

[17] Andreyev HJ, Norman AR, Cunningham D, Oates JR, Clarke PA (1998). Kirsten ras mutations in patients with colorectal cancer: The multicenter "RASCAL" study. J. Natl. Cancer Inst..

[18] Yoon HH (2014). KRAS codon 12 and 13 mutations in relation to disease-free survival in BRAF-wild-type stage III colon cancers from an adjuvant chemotherapy trial (N0147 alliance). Clin. Cancer Res..

[19] Taieb J (2017). Prognostic value of BRAF and KRAS mutations in MSI and MSS Stage III colon cancer. J. Natl. Cancer Inst..

[20] Etienne-Grimaldi MC (2014). Molecular patterns in deficient mismatch repair colorectal tumours: Results from a French prospective multicentric biological and genetic study. Br. J. Cancer.

[21] De Roock W (2010). Effects of KRAS, BRAF, NRAS, and PIK3CA mutations on the efficacy of cetuximab plus chemotherapy in chemotherapy-refractory metastatic colorectal cancer: A retrospective consortium analysis. Lancet Oncol..

[22] Sclafani F (2018). KRAS and BRAF mutations in circulating tumour DNA from locally advanced rectal cancer. Sci. Rep..

[23] Junca A (2020). Detection of colorectal cancer and advanced adenoma by liquid biopsy (Decalib Study): The ddPCR challenge. Cancers (Basel).

[24] Myint NNM (2018). Circulating tumor DNA in patients with colorectal adenomas: Assessment of detectability and genetic heterogeneity. Cell Death Dis..

[25] Thierry AR (2014). Clinical validation of the detection of KRAS and BRAF mutations from circulating tumor DNA. Nat. Med..

[26] Demuth C (2018). Measuring KRAS mutations in circulating tumor DNA by droplet digital PCR and next-generation sequencing. Transl. Oncol..

[27] Kruger S (2018). Repeated mutKRAS ctDNA measurements represent a novel and promising tool for early response prediction and therapy monitoring in advanced pancreatic cancer. Ann. Oncol..

[28] Charlson ME, Pompei P, Ales KL, MacKenzie CR (1987). A new method of classifying prognostic comorbidity in longitudinal studies: Development and validation. J. Chronic. Dis..

[29] Haller DG (2015). Impact of age and medical comorbidity on adjuvant treatment outcomes for stage III colon cancer: A pooled analysis of individual patient data from four randomized, controlled trials. Ann. Oncol..

